# Passivating Graphene and Suppressing Interfacial Phonon Scattering with Mechanically Transferred Large-Area Ga_2_O_3_

**DOI:** 10.1021/acs.nanolett.2c03492

**Published:** 2022-11-21

**Authors:** Matthew Gebert, Semonti Bhattacharyya, Christopher C Bounds, Nitu Syed, Torben Daeneke, Michael S. Fuhrer

**Affiliations:** † School of Physics and Astronomy, Monash University, Melbourne, Victoria 3800, Australia; ‡ ARC Centre of Excellence in Future Low-Energy Electronics Technologies, Monash University, Melbourne, Victoria 3800, Australia; § Leiden Institute of Physics, Leiden University, Niels Bohrweg 2, 2333 CA, Leiden, The Netherlands; ∥ School of Physics, The University of Melbourne, Parkville, Melbourne, Victoria 3010, Australia; ⊥ School of Engineering, RMIT University, Melbourne, Victoria 3000, Australia; # ARC Centre of Excellence in Future Low-Energy Electronics Technologies, RMIT University, Melbourne, Victoria 3000, Australia

**Keywords:** “chemical vapor deposition (CVD) graphene”, “mm-scale oxide dielectric”, “passivation”, “remote interfacial polar phonon scattering”, “van der Waals heterostructure”, “dielectric screening”

## Abstract

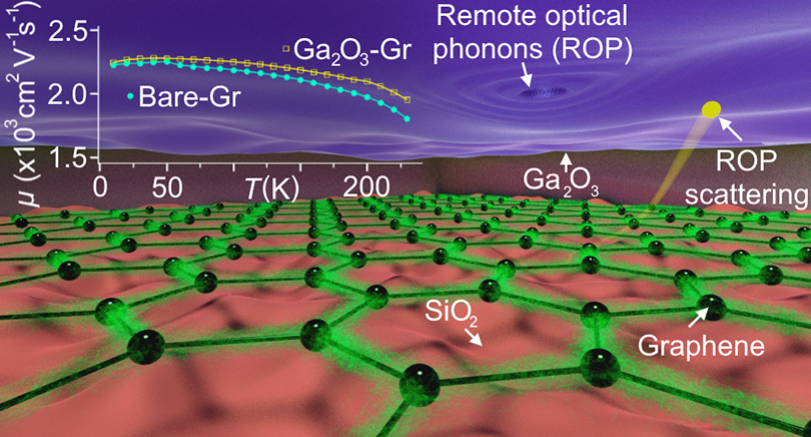

We demonstrate a large-area passivation layer for graphene by mechanical transfer of ultrathin amorphous Ga_2_O_3_ synthesized on liquid Ga metal. A comparison of temperature-dependent electrical measurements of millimeter-scale passivated and bare graphene on SiO_2_/Si indicates that the passivated graphene maintains its high field effect mobility desirable for applications. Surprisingly, the temperature-dependent resistivity is reduced in passivated graphene over a range of temperatures below 220 K, due to the interplay of screening of the surface optical phonon modes of the SiO_2_ by high-dielectric-constant Ga_2_O_3_ and the relatively high characteristic phonon frequencies of Ga_2_O_3_. Raman spectroscopy and electrical measurements indicate that Ga_2_O_3_ passivation also protects graphene from further processing such as plasma-enhanced atomic layer deposition of Al_2_O_3_.

Insulating layers are essential components of van der Waals heterostructures^1^–isolating materials electronically, passivating them, and enabling electrostatic gating. High-quality hexagonal boron nitride (h-BN), hand-exfoliated from small single crystals, has been widely used as a wide bandgap insulator for vdW heterostructures, enabling exceptional device quality.^2−5^

However, the growth of large area hBN is limited to mainly chemical vapor deposition growth on metal, which requires large-scale infrastructure, or high-purity costly metal rendering the process neither cost-effective nor user-friendly.^6−8^ In addition, hBN grown in this method can only be transferred on graphene through a wet-chemical process that can contaminate the heterostructure. This may cause difficulties with industrial-scale applications of hBN^9,10^ prompting a search for other suitable insulators to enable large-area vdW heterostructures.

In the case of encapsulating graphene, optimizing the material is highly complex; graphene’s electronic properties are largely determined by the insulator’s properties, including charged impurity concentration,^11^ dielectric constant,^12^ and surface optical (SO) phonons which remotely scatter carriers in the graphene,^13,14^ and trade-offs exist, e.g., insulators with intermediate dielectric constants may be optimal.^15^

Recently, the surface of liquid metals has been used to synthesize large-area atomically thin materials with facile mechanical transfer onto other substrates through a cost- and user-friendly process that does not require very high temperature, costly catalysts, toxic chemicals, or feedstock gases.^6,16−19^ Indeed, Ga_2_O_3_ has already been shown to be (i) an effective gate dielectric^20^ and (ii) an excellent encapsulating layer for transition metal dichalcogenide crystals (TMDs),^21^ preserving and even enhancing their optical properties.

Here we investigate liquid-metal synthesized Ga_2_O_3_ as a large-area encapsulating layer for graphene with an intermediate relative static dielectric constant κ ∼ 10.^22^ We mechanically transfer large-area (millimeter-scale) Ga_2_O_3_ onto one portion of a millimeter-scale graphene-on-SiO_2_ device, allowing us to compare the electronic transport properties of bare and Ga_2_O_3_-encapsulated portions of the same device. We find that coating graphene with Ga_2_O_3_ preserves the charge carrier mobility close to 3000 cm^2^ V^–1^ s^–1^. Surprisingly, we observe a reduction in temperature-dependent resistivity at temperatures below 220 K in the graphene encapsulated by the Ga_2_O_3_ dielectric, explained by the interplay of screening of the SO phonon modes of the SiO_2_ by high-dielectric-constant Ga_2_O_3_ and the relatively high characteristic phonon frequencies of Ga_2_O_3_ itself. We further show that Ga_2_O_3_ is useful as a passivation layer, protecting against damage from deposition of Al_2_O_3_ via plasma enhanced ALD.

Devices were fabricated (see Sections S1 and S2, Supporting Information) using a commercial (Graphene Supermarket^23^) CVD-grown monolayer graphene/monolayer h-BN film (henceforth referred to as “Gr”) already transferred onto a 285 nm SiO_2_/Si (p-doped) substrate that functions as a global back-gate dielectric and electrode. The Gr was then etched into a Hall bar geometry 0.4 mm wide and 1.2 mm long, with multiple voltage electrodes spaced by 0.25 mm, and contacted by Ti/Au electrodes fabricated using conventional photolithography. Next, millimeter-scale ultrathin Ga_2_O_3_ was prepared on a PPC film on a PDMS stamp (Gel-film, Gelpak) through a liquid metal “squeeze-printing”^19^ technique. Finally, Ga_2_O_3_ was deterministically transferred onto half of the Gr device.^21^ We compare the experimental signatures of “bare” and “Ga_2_O_3_-covered” parts of graphene in the same Hall bar device to understand the effect of Ga_2_O_3_.

Figure 1 illustrates the steps in the construction of the Ga_2_O_3_-on-Gr device. The process of transferring ultrathin Ga_2_O_3_ films on such Gr-devices is schematically represented in Figure 1a–d. First, a mm-scale ultrathin Ga_2_O_3_ film was prepared on a PPC film mounted on a PDMS stamp through a liquid metal printing technique known as “squeeze-printing”^19^ (Figure 1a). This film was then cut into approprate size to cover half of the Gr-device as well as to get rid of additional Ga-particles (Figure 1b), and was finally deterministically transferred onto half of the Gr Hall bar device using a homemade van der Waals stacking set up^21^ (Figure 1, parts c and d).

**Figure 1:**
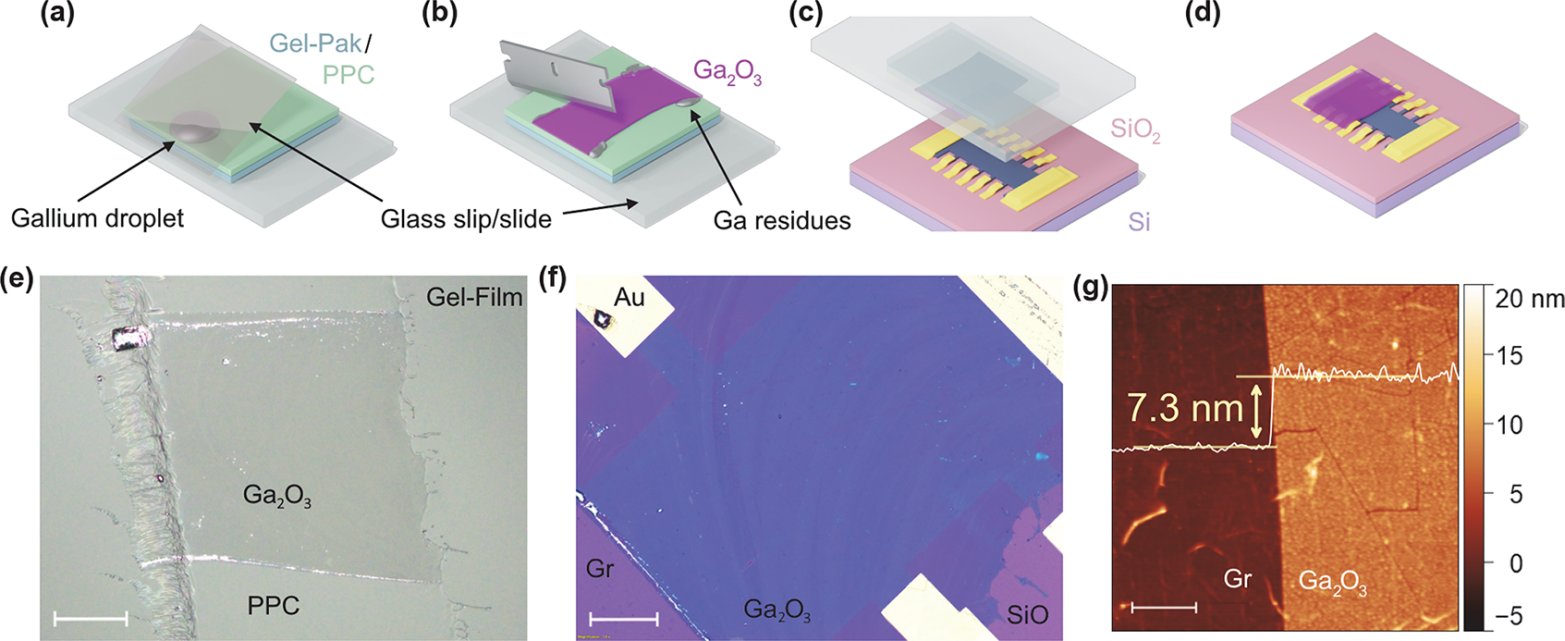
Characterization of Ga_2_O_3_ thin film transferred on Gr (CVD-grown monolayer graphene/monolayer h-BN film on SiO_2_). Schematic representation of (a) gallium metal positioned to be rolled across a PPC/Gel-Pak polymer stack using a coverslip, (b) Ga_2_O_3_ after rolling with some gallium metal residues, which can be cut away by a razor, (c) transfer of Ga_2_O_3_ film onto Gr/SiO_2_/Si device, and (d) device after removing polymer residues. (e) Optical darkfield micrograph of Ga_2_O_3_ on PPC after cutting to size. The silver colored dots are liquid gallium droplets. The Ga_2_O_3_ sheet at the center is bordered by liquid gallium. The scale bar is 200 μm. (f) Brightfield optical micrograph of Ga_2_O_3_ (deep blue sheet on the device) transferred on a Gr-device. The scale bar is 50 μm. (g) Topographic image of Ga_2_O_3_-on-Gr sheet obtained by intermittent contact atomic force microscopy (AFM). The left side of the image shows bare Gr, and right side of the image shows Ga_2_O_3_-covered Gr. Mean height difference is 7.3 nm as shown in the overlaid line profile, and the scalebar is 2 μm.

Figure 1e shows a dark-field image of a squeeze-printed Ga_2_O_3_ film on PPC/PDMS assembly. This film was trimmed to 0.7 mm × 0.65 mm to match the Gr-device. The darkfield image highlights the Ga-metal residue, left from the squeeze-printing processs, which is negligible in the interior area of the film, and mostly appears at the boundary. Figure 1f shows a bright-field optical image of the Ga_2_O_3_ film transferred on the Gr-device. The optical contrast of the amorphous Ga_2_O_3_ film is largely uniform, though slight variations are visible, indicating similar thickness across the thin film (Section S9, Supporting Information). Figure 1g shows an atomic force micrograph of both Ga_2_O_3_-covered (right-half) and bare side (left-half) of a Gr-device. The AFM line profile (overlaid) yields a step height of 7.3 nm for Ga_2_O_3_, similar to AFM measurements performed on similar devices (Section S9, Supporting Information), and consistent with thicknesses reported by Wurdack et al.^21^

Figure 2 compares the gate-voltage and temperature dependent electrical transport properties of the bare and Ga_2_O_3_-covered Gr-devices. The 3D schematic of the device and top-view micrograph are shown in Figure 2, parts a and b, respectively. Figure 2b shows that the transferred Ga_2_O_3_ film covers half of the Gr-device. The orientation of the Ga_2_O_3_ film has been carefully controlled so that the Ga-metal particles at the boundary do not affect the electrical transport characteristics between the voltage probes.

**Figure 2:**
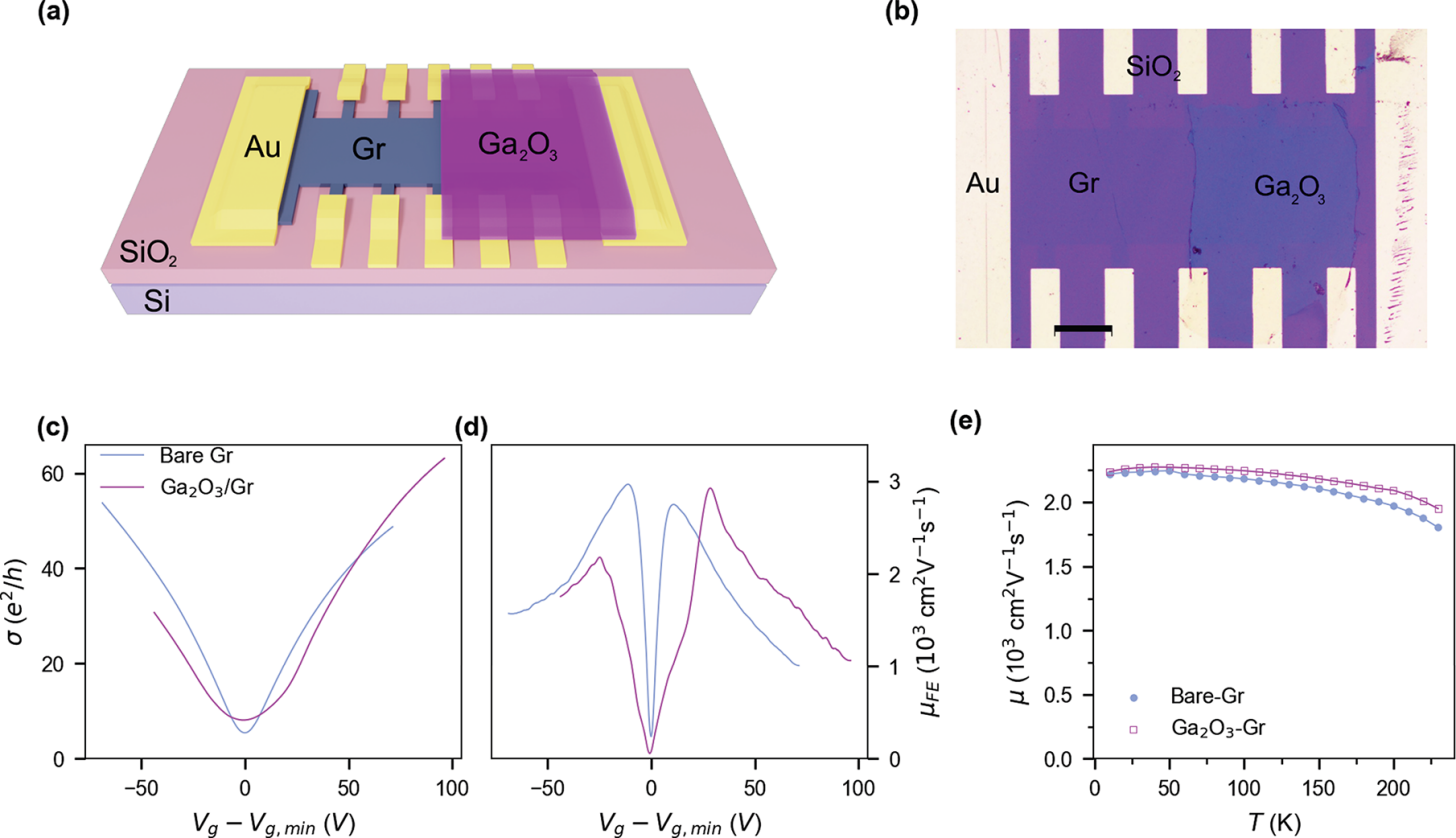
Gate voltage and temperature dependent electrical transport measurements of Ga_2_O_3_-covered and bare Gr field effect devices. (a) Schematic illustration and (b) optical microscope image of a Gr-device after Ga_2_O_3_ transfer. The scale bar is 200 μm. (c) Longitudinal conductivity σ and (d) field-effect mobility μ_FE_ as a function of gate voltage *V*_*g*_ offset by gate voltage at minimum conductivity *V*_*g,min*_, for both bare Gr and Ga_2_O_3_-covered Gr at 100 K. (e) Temperature- (*T*-) dependent effective mobility (μ) calculated at charge carrier density *n* = 5 × 10^12^ cm^–2^.

Figure 2c shows the gate voltage (*V*_*g*_) dependence of the longitudinal conductivity σ measured at temperature *T* = 100 K for both bare and Ga_2_O_3_-covered Gr. The gate voltage *V*_*g*_ is offset by the gate voltage of minimum conductivity (*V*_*g,min*_ = −1.2 and −26.2 V respectively for the bare and Ga_2_O_3_-covered sides) to facilitate comparison between the two sides of the device. The comparison of the two parts of the sample shows three notable differences, with the Ga_2_O_3_-covered part showing (i) slightly enhanced conductivity at high *V*_*g*_, (ii) increased magnitude of minimum conductivity σ_*min*_, and (iii) a broader minimum–conductivity plateau.

In order to highlight the first of these features we plot the field effect mobility (; *c*_*g*_ = capacitance of the SiO_2_ back gate) obtained from σ(*V*_*g*_) data (Figure 2d). The peak electron mobility is slightly improved in the Ga_2_O_3_-covered graphene (μ_FE_ = 2900 cm^2^ V^–1^ s^–1^) relative to bare graphene (μ_FE_ = 2800 cm^2^ V^–1^ s^–1^). The highest observed hole mobility in Ga_2_O_3_-covered Gr (μ_FE_ = 2200 cm^2^ V^–1^ s^–1^) is lower than in bare Gr (μ_FE_ = 3000 cm^2^ V^–1^ s^–1^) but might not be a global maximum, as it is at the edge of the *V*_*g*_ measurement window. μ_FE_ is observed to be mostly unchanged by the addition of Ga_2_O_3_, if not a little increased at high positive *V*_*g*_.

To understand whether the changes in mobility are due to Ga_2_O_3_ passivation and are reproducible, we have measured 60 additional devices, 30 with Ga_2_O_3_ passivation and 30 without; the detailed results are shown in Supporting Information, Section S11. We found that with the addition of Ga_2_O_3_, room temperature electron mobility μ_*e*_ increased by 47.7 ± 15.0% and hole mobility μ_*h*_ decreased by 52.2 ± 4.4% (See Supporting Information, Figure S6). Due to the greater importance of remote optical phonon scattering at room temperature (see below) a quantitative comparison is difficult, but the room-temperature observations are qualitatively similar to that seen in Figure 2d at 100 K. Control devices processed identically but without Ga_2_O_3_ passivation showed smaller changes (3.8 ± 12.1% decrease in μ_*e*_ and 18.4 ± 7.9% increase in μ_*h*_) which are likely due to cleaning and annealing.

The slight enhancement of mobility after deposition of Ga_2_O_3_ is remarkable because previous experiments found that deposition of oxide usually degrades the mobility of graphene^24^ due to introduction of disorder. In contrast, screening by a clean dielectric can reduce charged impurity scattering.^12,25^ To infer the impurity concentration in SiO_2_/Gr/Ga_2_O_3_ with RPA-Boltzmann theory,^26^ we use κ = 10 for the amorphous Ga_2_O_3_^22^ and μ = 2900 cm^2^ V^–1^ s^–1^ and calculate an impurity concentration of *n*_*imp*,Ga_2_O_3__ = 3.8 × 10^12^ cm^–2^ (Section S6.A in the Supporting Information), roughly twice the impurity concentration inferred for our bare graphene on SiO_2_ (*n*_imp,bare_ = 1.8 × 10^12^ cm^–2^). This indicates that our liquid-metal synthesized and mechanically transferred Ga_2_O_3_ layer has low charged impurity concentration, comparable to thermally grown SiO_2_.

According to the RPA-Boltzmann theory, the increased *n*_*imp*_ should also lead to a very weak reduction of σ_*min*_^26^ and a narrowing of the minimum conductivity plateau in the Ga_2_O_3_-covered graphene (Section S6.B in the Supporting Information), in contrast to our observation (Figure 2c). The increased σ_*min*_, as well as broadening of the minimum conductivity plateau, likely instead reflects additional macroscopic inhomogeneity of the sample^27^ induced by the Ga_2_O_3_.

In order to further explore the modification of electrical transport in Ga_2_O_3_-covered graphene, we plotted the effective mobility μ =  calculated at *n* = 5 × 10^12^ cm^–2^ for both bare and Ga_2_O_3_-covered graphene (Figure 2e). We observe a gradual reduction in mobility with increasing temperature between 60 and ≈220 K in both. The overall decline of mobility indicates a temperature-dependent resistivity contribution, which surprisingly appears larger in bare compared to Ga_2_O_3_-covered graphene, in contrast to previous experiments where addition of an oxide layer on graphene increased the temperature-dependent resistivity.^28^

We expect that dielectric layers affect the temperature dependent mobility of graphene through scattering of charge carriers by SO phonons. This process, also known as remote optical phonon (ROP) scattering, is expected to contribute a resistivity proportional to a Bose–Einstein distribution.^14^
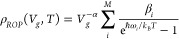
1Here the *i*th SO mode is described by the SO phonon energy ℏω^*i*^ (meV) and respective coupling strength β_*i*_ (*V*_*g*_^α^). Empirically, the dependence on gate voltage is found to follow a power law with α ≈ 1.

To examine the differences in ROP scattering for bare and Ga_2_O_3_-covered graphene, we extracted ρ_*ROP*_(*V*_*g*_,*T*) from ρ(*V*_*g*_,*T*) (see Section S10, Supporting Infromation for details). Briefly, we perform a global fit of ρ(*V*_*g*_,*T*) at different *V*_*g*_ at 70 K ≤ *T* ≤ 100 K to determine the acoustic phonon scattering contribution ρ_*LA*_(*T*), which is linear in temperature and independent of *V*_*g*_, and the impurity contribution ρ_*imp*_(*V*_*g*_, *T* = 0 K), which depends on *V*_*g*_ but not temperature. Subtracting these two quantities from ρ(*V*_*g*_,*T*) allows us to extract ρ_*ROP*_(*V*_*g*_, *T*).

Figure 3a shows the contribution of ROP scattering ρ_*ROP*_(*V*_*g*_, *T*) to resistivity as a function of temperature, for various positive gate voltages (offset from the minimum *V*_*g,min*_). ρ_*ROP*_(*V*_*g*_, *T*) increases superlinearly in temperature, with larger magnitude at smaller *V*_*g*_ – *V*_*g,min*_. Remarkably, in the temperature range 70–220 K, ρ_*ROP*_ is lower in the Ga_2_O_3_-covered Gr compared to bare Gr for all values of *V*_*g*_ – *V*_*g,min*_. The dashed lines in Figure 3a are global fits of the data to eq 1 for a single phonon mode (*M* = 1) for bare (dashed) and Ga_2_O_3_-covered (dot-dash) graphene.

**Figure 3:**
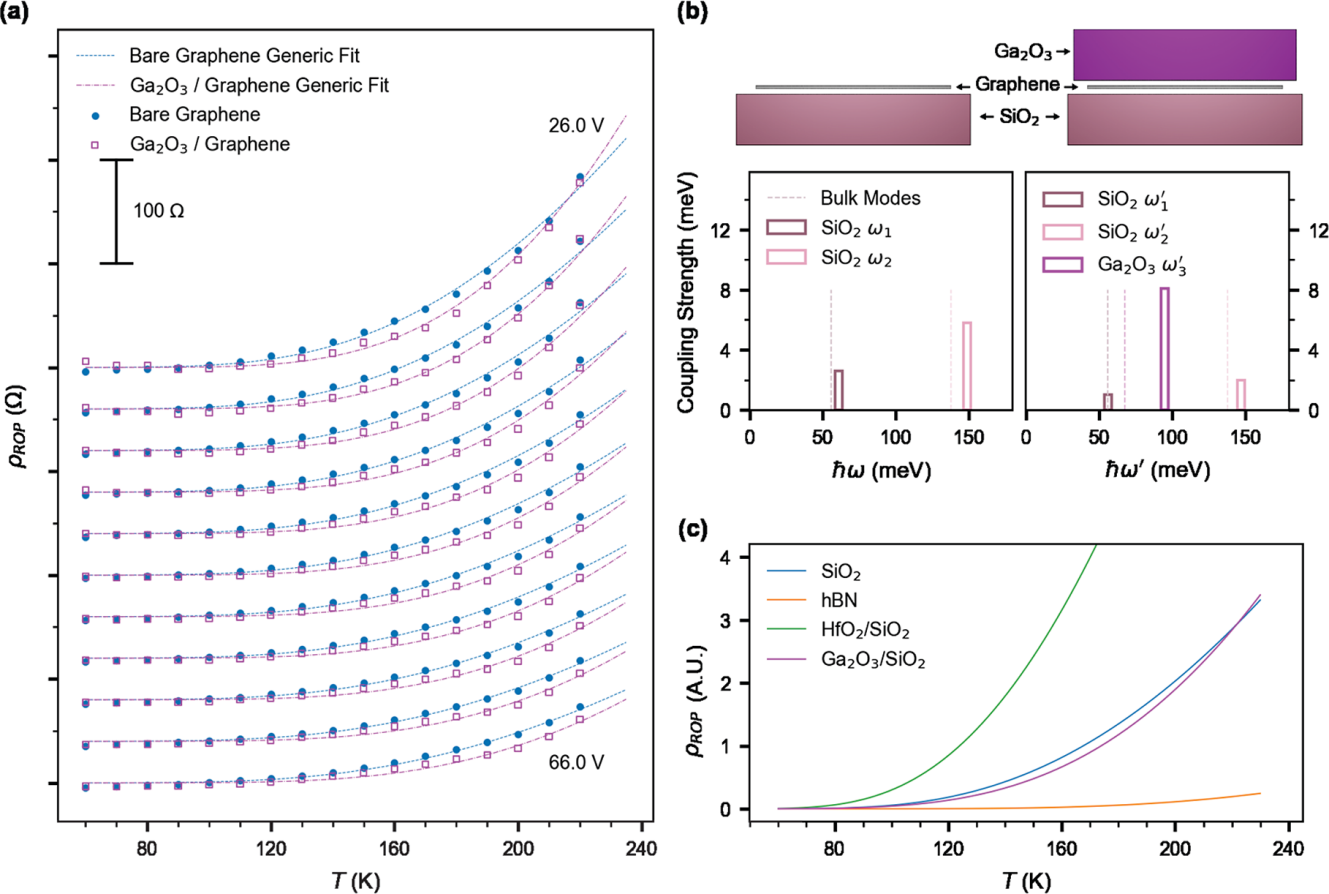
Remote optical phonon (ROP) scattering. (a) ROP contributions to resistivity (ρ_*ROP*_) extracted from the temperature- (*T*-) dependence of resistivity (ρ) in bare (filled) and Ga_2_O_3_-covered (hollow) graphene. Fits using eq 1 are plotted with dashed line for bare and dot-dashed line Ga_2_O_3_-covered Gr. For each gate voltage, from 26 through 66 V via 4 V steps, ρ_*ROP*_ is offset by 40 Ω. (b) Computed frequency and coupling strength of SO phonons in bare and Ga_2_O_3_-covered Gr. Dashed lines indicate the corresponding bulk mode phonon frequency. (c) Modeled ROP scattering contribution to the resistivity (ρ_*ROP*_) in graphene for different dielectric systems.

The results of the fits in Figure 3a are summarized in Table 1. We determine the SO mode energy, ℏω_0_, to be larger in Ga_2_O_3_-covered graphene (92.8 meV) compared to bare graphene on SiO_2_ (69.5 meV). The observation of slightly higher ℏω_0_ compared to the expected lowest phonon mode for SiO_2_ (61 meV) was also observed by Chen et al.,^14^ and it is likely due to the additional contribution of the higher-energy SiO_2_ mode. The power-law exponent α is similar for bare and Ga_2_O_3_-covered graphene and close to that of the previous literature.^14,28^ The coupling strength β is found to be 6.0 (*h*/*e*^2^) for bare graphene, which is roughly double the value of 3.26 (*h*/*e*^2^) found by Chen et al.^14^ Due to the different values of α for Ga_2_O_3_-covered graphene, it is difficult to directly compare the coupling β, which has different dimensions and consequently has a different magnitude to that for bare graphene.

**Table 1:** Parameters for Fits of Data in Figure 3 for Bare and Ga_2_O_3_-Covered Graphene to Equation 1

	α	β (V^–α^ *h*/*e*^2^)	ℏω_0_ (meV)
Bare	0.97 ± 0.03	6.0 ± 1.1	69.5 ± 1.3
Ga_2_O_3_	1.18 ± 0.03	43.1 ± 6.7	92.8 ± 2.6

Our observations (Table 1) indicate that the smaller magnitude of ρ_*ROP*_(*V*_*g*_, *T*) at low temperatures (*T* ≲ 220 K) for Ga_2_O_3_-covered graphene is due to a higher effective phonon energy, resulting in a lower ρ_*ROP*_(*V*_*g*_, *T*) at low temperatures due to lower phonon population, and eventually crossing over to higher ρ_*ROP*_(*V*_*g*_, *T*) at high *T* due to stronger coupling. To better understand the lower ρ_*ROP*_ contribution in Ga_2_O_3_-covered graphene, we develop a simple analytical model of the SiO_2_/graphene/Ga_2_O_3_ heterostructure, following the methodology used in semiconductor inversion layers^29^ and graphene systems.^13,28^ The details of the model are described in Section S8 of the Supporting Information.

The resultant phonon frequencies and coupling constants of both bare and Ga_2_O_3_-covered graphene is schematically represented in Figure 3b (see Tables S2 and S3–S6, Supporting Information, for more details). We find that the graphene/SiO_2_ structure has two SO modes, i.e., ℏω_1_ = 61 meV and ℏω_2_ = 149 meV. The Ga_2_O_3_/graphene/SiO_2_ structure has three SO modes, with energies and coupling strengths shown in Figure 3b. Here ℏω_1_^′^ = 56 meV, ℏω_2_^′^ = 147 meV are the perturbed SiO_2_ modes, while ℏω_3_^′^ = 95 meV originates in Ga_2_O_3_. While all these modes are thermally activated, the Ga_2_O_3_ mode couples particularly strongly, also reflected in the high disparity between ϵ_*Ga*_2_*O*_3__^0^ and ϵ_*Ga*_2_*O*_3__^∞^ (Table S2 and Section S8, Supporting Information). At the same time, however, the large ϵ_*Ga*_2_*O*_3__^∞^ screens the ROP scattering from SiO_2_ modes. Hence we expect the ω_3_^′^ mode to dominate the temperature-dependent resistivity. Importantly, the energy corresponding to the ω_3_^′^ mode matches well with the ℏω_0_ value obtained from fitting our experimental data (Figure 3a) and Table 1.

Figure 3c shows the analytically obtained ρ_*ROP*_(*T*) for the Ga_2_O_3_/graphene/SiO_2_ structure and bare graphene/SiO_2_ calculated using eq 1, using the SO modes as shown in Figure 3c (also given in Table S3 and Table S6, Supporting Information). Also shown for comparison are graphene/h-BN, and HfO_2_/graphene/SiO_2_ (Tables S4 and S5, Supporting Information). We see that ρ_*ROP*_(*T*) in Ga_2_O_3_/graphene/SiO_2_ is lower than for bare graphene/SiO_2_ at temperatures below approximately 220 K. Because ROP is thermally activated, at low temperatures the SiO_2_ mode (ω_1_) dominates ρ_*ROP*_ for bare graphene/SiO_2_, while for the Ga_2_O_3_/graphene/SiO_2_ structure, the Ga_2_O_3_ effectively screens these SiO_2_ contributions. At higher *T*, the Ga_2_O_3_ ROP scattering with higher mode energy (ω_3_^′^ > ω_1_^′^) becomes active and quickly begins to dominate due to the higher coupling. Our observation that addition of Ga_2_O_3_ to graphene/SiO_2_ can lower the overall interfacial phonon scattering may inspire the design of other heterostructures to further reduce scattering phenomena of charge carriers in graphene, perhaps yielding a more significant improvement at or above room temperature.

In our model, we have ignored the effect of the monolayer h-BN in between graphene and SiO_2_. The experimental data agree well with our model, and this is consistent with previous ROP experimental models in graphene on SiO_2_.^14,28^ This might be surprising because one might expect the hBN monolayer crystal to play a significant role in determining the ROP scattering in graphene. However, the experimental results suggest otherwise. These results suggest that the surface modes of h-BN/SiO_2_ are comparable to that of bare SiO_2_; however, further work is needed to understand why that is the case. This may be explained by the similar dielectric constants of the SiO_2_ and h-BN resulting in similar SO properties for the monolayer h-BN/SiO_2_ composite to bare SiO_2_. Our model on SiO_2_/graphene/HfO_2_ device matches with previous results as expected.^28^

Having demonstrated that Ga_2_O_3_ does not enhance impurity scattering in graphene and even reduces the impact of phonon scattering in a certain temperature range, we now investigate whether Ga_2_O_3_ is effective in protecting graphene from further processing. Methods of growing large-area dielectric films, such as CVD, atomic layer deposition (ALD), sputtering, and e-beam evaporation, have proven to be damaging for graphene, leading to enhancement of impurity scattering and consequently degradation of mobility.^28,30−34^

Our gentle transfer technique for thin Ga_2_O_3_ avoids such damaging processes, as demonstrated in Figure 2. We further demonstrate the protective nature of Ga_2_O_3_ by using plasma-enhanced ALD to grow a 5.5 nm layer of Al_2_O_3_ over the entire sample, following the method of Tang et al.^31^ (see Section S3, Supporting Information, for further details), in order to replicate conditions that normally might damage graphene. The ALD chamber temperature is modified to 150 °C, to avoid possible change of morphology of amorphous Ga_2_O_3_ by thermal annealing.^21^

Figure 4 compares the effect of Al_2_O_3_ deposition on the bare and the Ga_2_O_3_-covered side of the Gr device. Parts a and b of Figure 4 show Raman spectra on bare and Ga_2_O_3_-covered sides respectively before and after ALD deposition. Both areas of the device show monolayer graphene G and 2D Raman peaks at 1591 and 2709 cm^–1^, with expected ratio ∼2. Before ALD deposition the graphene spectra on both sides are nearly identical, confirming that Ga_2_O_3_-transfer process does not lead to any structural disorder in graphene. However, there is a stark difference between the Raman spectra on both sides of the devices after ALD processing, where the bare graphene area shows a fully formed D-peak (1357 cm^–1^), which is nearly absent in preprocessed graphene and remains unchanged in the Ga_2_O_3_-covered graphene even after processing. The D peak is activated by point disorder and indicates deposition-induced damage in bare graphene, which is not present in Ga_2_O_3_-covered graphene. This clearly indicates that the Ga_2_O_3_ transfer has no adverse effect on graphene, and it also acts as an encapsulating layer to protect graphene against further damage during subsequent deposition processes.

**Figure 4:**
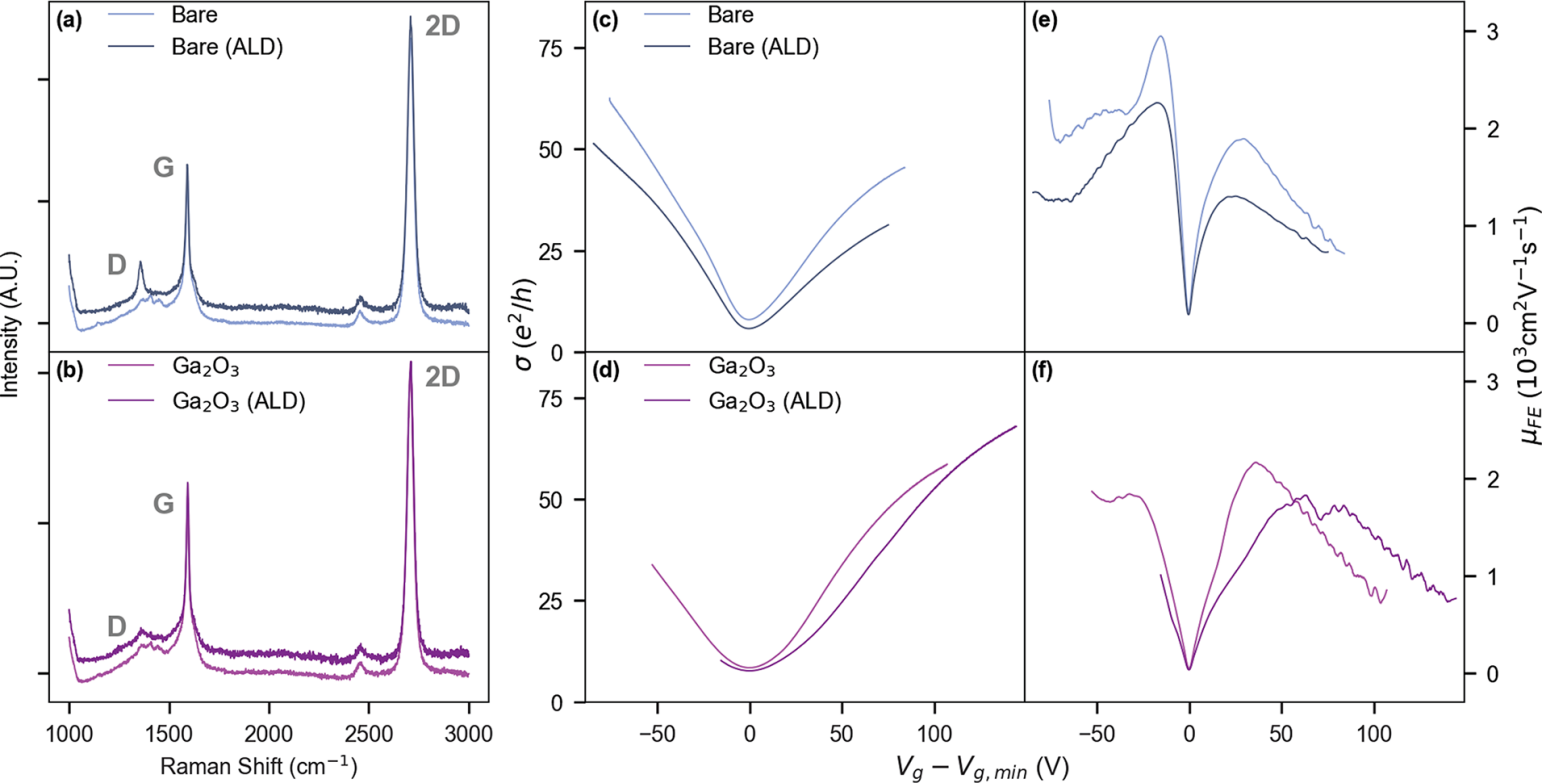
Ga_2_O_3_ as a protective layer on graphene against plasma-enhanced atomic layer deposition (ALD) of Al_2_O_3_. Raman spectroscopy with D, G, and 2D peaks indicated for (a) bare graphene and (b) Ga_2_O_3_-covered graphene. Gate-dependence of conductivity (σ) and field-effect mobility (μ_*FE*_) respectively for (c, e) bare Gr and (d, f) Ga_2_O_3_-covered Gr. Data is shown for the same samples before (lighter shade) and after (darker shade) of the ALD process. Raman and transport data are taken from different samples at room temperature.

This is further supported through electrical transport data obtained on both sides of an identically prepared device before and after Al_2_O_3_ deposition (Figure 4c–f). Figure 4c,d and Figure 4e,f show the relative change in σ and μ_*FE*_ respectively before and after ALD, both on bare and Ga_2_O_3_-covered graphene. After ALD processing, there is a global decrease in σ and μ_*FE*_ for bare graphene, and peak μ_FE_ drops by ≈30%, compared to >15% in Ga_2_O_3_-covered graphene. At higher gate voltages (*V*_*g*_ – *V*_*g,min*_ > 50 V) the transport in processed Ga_2_O_3_-covered graphene shows similar performance to prior to ALD, with higher μ_*FE*_ values, and similar σ at ≈100 V. The broadening (and slight reduction) of the μ_*FE*_ peak in the Ga_2_O_3_-covered side (Figure 4f) appears to have been caused by further enhancement of the inhomogeneity already present in the system, as the higher mobility at high carrier density indicates that ALD on Ga_2_O_3_-covered graphene does not induce additional impurities, in agreement with the Raman spectroscopy results.

We checked the reproducibility of Ga_2_O_3_ passivation as a protective layer using the 60 additional devices previously discussed (Supporting Information, Section S11). After deposition of Al_2_O_3_ via ALD on Ga_2_O_3_ passivated and bare devices, we find that the Ga_2_O_3_ passivation provided significant protection of mobility for both electron (12.0 ± 11.0% increase) and hole carriers (5.6 ± 13.6% increase; see Supporting Information, Figure S7). After ALD, the bare devices underwent a large reduction in hole mobility (42.7 ± 7.9%), while electron mobility varied greatly (net 16.9 ± 28% increase), indicating more disorder compared to Ga_2_O_3_-covered samples, similar to the effects seen in Figure 2.

Our results demonstrate that liquid-metal synthesized Ga_2_O_3_ is a viable large-area mechanically transferred passivation layer for vdW heterostructures. Encapsulation of graphene by Ga_2_O_3_ preserves the mobility, and reduces ROP scattering in graphene below *T* = 220 K due to the interplay of high energy phonon modes and dielectric screening in this oxide with intermediate dielectric constant. The large area passivation capability of Ga_2_O_3_ enables other deposition methods without causing damage at the interface, which should allow integration with a variety of materials and processes. The liquid metal printing technique is highly versatile with a wide range of materials already demonstrated;^19^ hence, this work opens the possibility of expanding to other liquid metal printed ultrathin materials for large-area vdW heterostructures.

## Supporting Information Available

Device fabrication, mechanical transfer of Ga_2_O_3_, atomic layer deposition (ALD) of Al_2_O_3_, electrical measurements in PPMS and probe station, Raman spectroscopy, self-consistent theory for graphene’s conductivity, temperature-dependence of the hysteresis of σ–*V*_g_ in bare graphene and Ga_2_O_3_-covered graphene, remote optical phonon (ROP) scattering model, Ga_2_O_3_ atomic-force and darkfield microscopy, extraction of ρ_*ROP*_ as a function of temperature, additional devices to assess reproducibility of changes in transport properties after Ga_2_O_3_ passivation and ALD, and Figures S1–S8 (PDF)
