# Supplementary material for: Passivating Graphene and Suppressing Interfacial Phonon Scattering with Mechanically Transferred Large-Area Ga2O3
Source: Nano Lett. 2022 Nov 21;23(1):363–70. doi: 10.1021/acs.nanolett.2c03492 (PMC9837877; doi:10.1021/acs.nanolett.2c03492)
Supplement: Supplementary file 1 — nl2c03492_si_001.pdf [file nl2c03492_si_001.pdf]

Supporting Information

Mechanically transferred large-area Ga_2O_3 passivates graphene and suppresses interfacial phonon scattering

Matthew Gebert,[1,2] Semonti Bhattacharyya,[1,2,3,*] Christopher C Bounds,[1] Nitu Syed,[4,5] Torben Daeneke,[5,6] and Michael S. Fuhrer[1,2,*]

[1] *School of Physics and Astronomy, Monash University, Victoria 3800, Australia*
[2] *ARC Centre of Excellence in Future Low-Energy Electronics Technologies, Monash University, Victoria 3800 Australia*
[3] *Leiden Institute of Physics, Leiden University, Niels Bohrweg 2, 2333 CA, Leiden, The Netherlands*[†]
[4] *School of Physics, The University of Melbourne, Melbourne, Parkville VIC 3010, Australia*
[5] *School of Engineering, RMIT University, Melbourne, Victoria 3000, Australia*
[6] *ARC Centre of Excellence in Future Low-Energy Electronics Technologies, RMIT University, Melbourne, Australia 3000*

(Dated: November 15, 2022)

Contents

S1. Device Fabrication 1
S2. Mechanical transfer of Ga_2O_3 1
S3. Atomic Layer Deposition (ALD) of Al_2O_3 3
S4. Electrical Measurements in PPMS & Probestation 3
S5. Raman Spectroscopy 3
  A. Electrical Analysis 3
S6. Self-consistent theory for graphene's conductivity 4
  A. Impurity concentration 5
    1. Bare graphene on SiO_2 5
    2. Ga_2O_3-covered graphene on SiO_2 5
  B. Change of n^* and σ_{min} in Ga_2O_3-covered graphene 5
S7. Temperature-dependence of the hysteresis of σ-V_g in bare graphene and Ga_2O_3-covered graphene 7
S8. Remote optical phonon (ROP) Scattering Model 8
S9. Ga_2O_3 Atomic-force & Darkfield Microscopy 10
S10. Extracting ρ_{ROP} as a function of temperature 12
S11. Additional devices to assess reproduciblity of changes in transport properties after Ga_2O_3 passivation and ALD 14
  A. Device fabrication and general properties 14
  B. Statistical analysis 16
    1. Ga_2O_3 transfer 16
    2. ALD Al_2O_3 deposition 17
S12. References 18

S1. Device Fabrication

Graphene/h-BN/SiO_2/Si heterostructures were commercially purchased from Graphene Supermarket, consisting of 1 cm square monolayer graphene on monolayer h-BN, both wet transferred onto 285 nm SiO_2 on 525 μm Si (100) p-doped substrates. This heterostructure was etched into Hall bars using an argon-oxygen plasma, via standard photolithographic techniques and a PVA TePla Plasma Asher.

S2. Mechanical transfer of Ga_2O_3

We started with a PPC mask prepared by spin coating PPC (Polypropylene carbonate) onto Gel-Film from Gel-Pak at 3000 rpm, and baked at 100 °C. Gallium was heated to liquid phase using a hotplate at 40 °C in ambient. A drop of gallium was then placed on the PPC mask at 40 °C. The liquid metal was then "rolled" by flattening a glass coverslip on the surface, depositing the Ga_2O_3 sheets on the PPC. Followed by this, the polymer stack consisting of Gel-film, PPC, and Ga_2O_3 was cut for sizing and to remove excess PPC & gallium metal residues using a hot scalpel. Finally, PPC & Ga_2O_3 stack was transferred onto the device using a remote-controlled micro-mechanical transfer apparatus with a microscope to align to device geometry. Transfer occurred at temperatures up to 120 °C. PPC was removed by dissolving it in acetone. A schematic of this process is illustrated at Fig. S1 below.

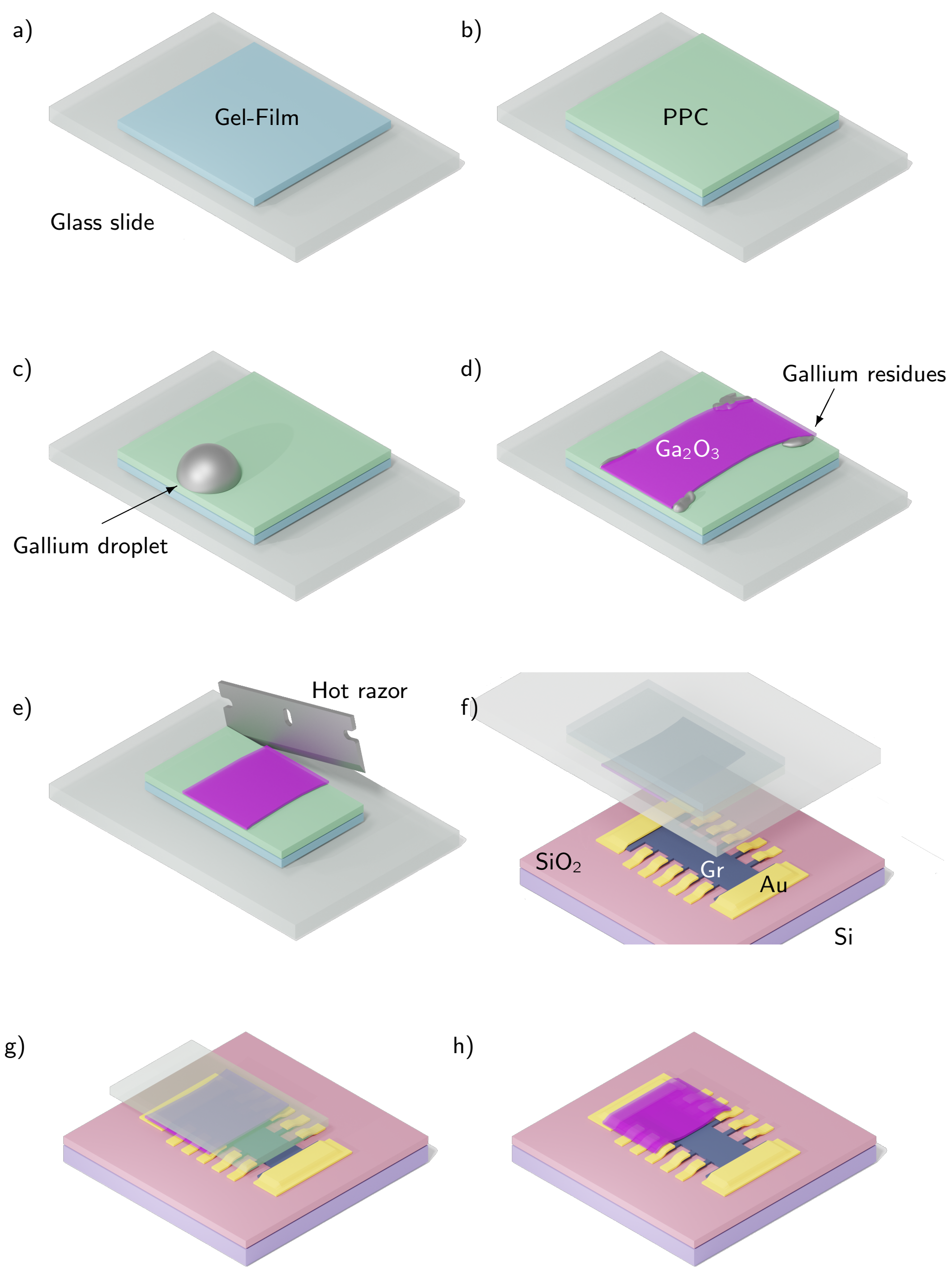

FIG. S1: Transfer process for Ga_2O_3.
(a) Gel-Film on glass slide. (b) PPC spin-coated onto gel film. (c-d) Gallium metal rolled over PPC to deposit Ga_2O_3. (e) Gel-Film/PPC/Ga_2O_3 stack cut using high temperature razor. (f) Gel-Film/PPC/Ga_2O_3 stack flipped to transfer onto SiO_2/Graphene device using a transfer stage with a microscope. (g) PPC/Ga_2O_3 deposited by heating the sample to 120°C. (h) PPC washed in Acetone/IPA leaving clean.

S3. Atomic Layer Deposition (ALD) of Al_2O_3

Al_2O_3 ALD was performed following the plasma-enhanced methodology of Tang *et al.*,[1] using a Fiji F200 ALD instrument from Ultratech/Cambridge NanoTech Inc. The metallic precursors were released first, prior to oxidant precursor created through plasma exposure, to minimize plasma damage to the graphene surface in the first cycle. The ALD temperature differed from Tang *et al.*, being performed at 150 °C instead of 250 °C, in order to keep annealing conditions consistent for Ga_2O_3.[2] 55 cycles were applied, leading to a growth of 5.5 nm thick Al_2O_3-film as confirmed on Si reference samples by ellipsometry.

S4. Electrical Measurements in PPMS & Probestation

Samples were wirebonded to chip carriers and measured using a Quantum Design PPMS 6000, operating between 4 K and 400 K under vacuum (< 10 mTorr) in presence of He exchange gas. Samples were annealed first for 8 hours at 390 K in vacuum.

ALD-processed samples were measured in a Lakeshore TTPX probestation at room temperature, with vacuum $\approx 10^{-4}$ mBar, both before and after ALD. Annealing was also performed in these samples for 8 hours at 390 K.

Four-probe resistance was measured using a standard lock-in technique, using two Stanford Research SR830 lock-in amplifiers to synchronously measure the voltages across both bare and Ga_2O_3 areas of Gr. Low current (100 nA) was supplied as a current source through the devices to avoid Joule heating. The highly-doped Si-substrate was used as a backgate with voltage applied through a Keithley 2400 sourcemeter.

S5. Raman Spectroscopy

Raman spectroscopic measurements were acquired at room temperature using a Renishaw InVia microscope, using a blue 488 nm source with a 50X objective. Data was taken across at least 10 points in a sample. A quadratic baseline was subtracted from each spectrum. All curves were normalised to the G peak amplitude in order to facilitate comparison between different samples.

A. Electrical Analysis

To calculate the field-effect mobility, the $\rho(V_g)$ data was first smoothed using a Savitzky-Golay filter to remove discretisation at high carrier density. This was then differentiated, followed by application of a second Savitzky-Golay filter.

S6. Self-consistent theory for graphene's conductivity

Graphene's conductivity on SiO_2 is calculated using self-consistent theory from Adam *et al.*.[3] At high carrier density $n > n^*$, the residual carrier density, graphene's conductivity σ is given by:

$$\sigma = \frac{2e^2}{h}\frac{n}{n_{imp}}\frac{1}{G\left[r_s\right]} \tag{S1}$$

$$\frac{\pi}{4} + 3x - \frac{3\pi x^2}{2} \tag{S2}$$

$$+ \frac{x\left(3x^2-2\right)\arccos\left[1/x\right]}{\sqrt{x^2-1}} \tag{S3}$$

where n_{imp} is the impurity density, and r_s is the characteristic interaction length (Wigner Seitz radius), calculated for a local environment dielectric of strength $\epsilon \equiv \kappa\epsilon_0$ where ϵ_0 is the electric constant:

$$r_s\left[\kappa\right] = \frac{1}{4\pi\epsilon_0}\frac{e^2}{\kappa}. \tag{S4}$$

κ is the average of relative dielectric constants of the layers above and below graphene

$$\kappa \sim \frac{\kappa_1 + \kappa_2}{2} \tag{S5}$$

$$\tag{S6}$$

which gives the average relative dielectric constants for bare-Gr and Ga_2O_3-Gr,

$$\kappa_{\mathrm{SiO_2/Bare}} \sim \frac{3.9 + 1.0}{2} = 2.5, \tag{S7}$$

$$\kappa_{\mathrm{SiO_2/Ga_2O_3}} \sim \frac{4.0 + 10.0}{2} = 7.0, \tag{S8}$$

and r_s for bare-Gr and Ga_2O_3-Gr,

$$r_s^{\mathrm{SiO_2/Bare}} = 0.8, \tag{S9}$$

$$r_s^{\mathrm{SiO_2/Ga_2O_3}} = 0.3. \tag{S10}$$

Hence, using Eq. (S1) we can deduce σ for bare-Gr and Ga_2O_3-Gr,

$$\sigma_{\mathrm{SiO_2/Bare}} = 20\frac{e^2}{h}\frac{n}{n_{imp}^{\mathrm{SiO_2/Bare}}}, \tag{S11}$$

$$\sigma_{\mathrm{SiO_2/Ga_2O_3}} = 45\frac{e^2}{h}\frac{n}{n_{imp}^{\mathrm{SiO_2/Ga_2O_3}}}. \tag{S12}$$

A. Impurity concentration

To calculate the impurity concentration for each substrate, we can use a self-consistent RPA method.[3]

1. *Bare graphene on SiO$_2$*

For bare graphene , the conversion from mobility to impurity density is already calculated by Adam *et al.*:[3]

$$\frac{\mu_0}{\mu_{\mathrm{SiO_2/Bare}}} \sim 50 \frac{n_0}{n_{imp}^{\mathrm{SiO_2/Bare}}} \tag{S13}$$

where $\mu_0 = 10,000$ cm^2/Vs and $n_0 = 10^{10}$ cm^{-2}. Using an electron mobility of 2,800 cm^2/Vs, the impurity density is inferred to be

$$n_{imp}^{\mathrm{SiO_2/Bare}} = 1.8 \times 10^{12}\ \mathrm{cm}^{-2}. \tag{S14}$$

2. *Ga$_2$O$_3$-covered graphene on SiO$_2$*

As the prefactor is different in eqn.S11 and eqn.S12 for Ga$_2$O$_3$-covered graphene, Eq. (S13) changes to become:

$$\frac{\mu_0}{\mu_{\mathrm{SiO_2/Ga_2O_3}}} \sim 110 \frac{n_0}{n_{imp}^{\mathrm{SiO_2/Ga_2O_3}}}. \tag{S15}$$

Using the mobility of the Ga$_2$O$_3$ covered area, 2900 cm^2/Vs,

$$n_{imp}^{\mathrm{SiO_2/Ga_2O_3}} = 3.8 \times 10^{12}\ \mathrm{cm}^{-2}. \tag{S16}$$

B. Change of n^* and σ_{min} in Ga$_2$O$_3$-covered graphene

According to the self-consistent theory by Adam *et al.*[3] the minimum conductivity σ_{min} in bare graphene on SiO$_2$ is

$$\sigma_{min}^{\mathrm{SiO_2/Bare}} = 20 \frac{e^2}{h} \frac{n^*_{\mathrm{SiO_2/Bare}}}{n_{imp}^{\mathrm{SiO_2/Bare}}} \tag{S17}$$

where n^*, the residual carrier density, is proportional to the δV, the width of the minimum conductivity plateau.

Similarly, for Ga$_2$O$_3$-covered graphene on SiO$_2$

$$\sigma_{min}^{\mathrm{SiO_2/Ga_2O_3}} = 45 \frac{e^2}{h} \frac{n^*_{\mathrm{SiO_2/Ga_2O_3}}}{n_{imp}^{\mathrm{SiO_2/Ga_2O_3}}}. \tag{S18}$$

n^* can be determined from numerically solving the following self-consistent equation,

$$\frac{n^*}{n_{imp}} = 2 r_s^2 C_0^{RPA}(r_s, a = 4d\sqrt{\pi n^*}). \tag{S19}$$

where

$$C_0^{RPA}(r_s, a) = -1 + \frac{4E_1(a)}{(2+\pi r_s)^2} + \frac{2e^{-a}r_s}{1+2r_s}$$
$$+(1+2r_s a)e^{2r_s a}(E_1[2r_s a] - E_1[1+2r_s a])$$

and

$$E_1(Z) = [\int_z^\infty t^{-1} e{-}t\, dt] \quad \text{(S20)}$$

We obtain the theoretically expected values for n^* and σ_{min} for SiO_2 and Ga_2O_3 using eqn. S19 and n_{imp} obtained in section S2.A. The results are shown in Table S1 below.

TABLE S1: Theoretically expected n^* and σ for bare graphene and Ga_2O_3-covered graphene

Dielectric environment	n_{imp} (cm^{-2})	n^* (cm^{-2})	σ_{min} (e^2/h)
Bare graphene on SiO_2	1.8e12	4.4e11	4.9
Ga_2O_3-covered graphene on SiO_2	3.8e12	3.9e11	4.6

S7. Temperature-dependence of the hysteresis of σ-V_g in bare graphene and Ga_2O_3-covered graphene

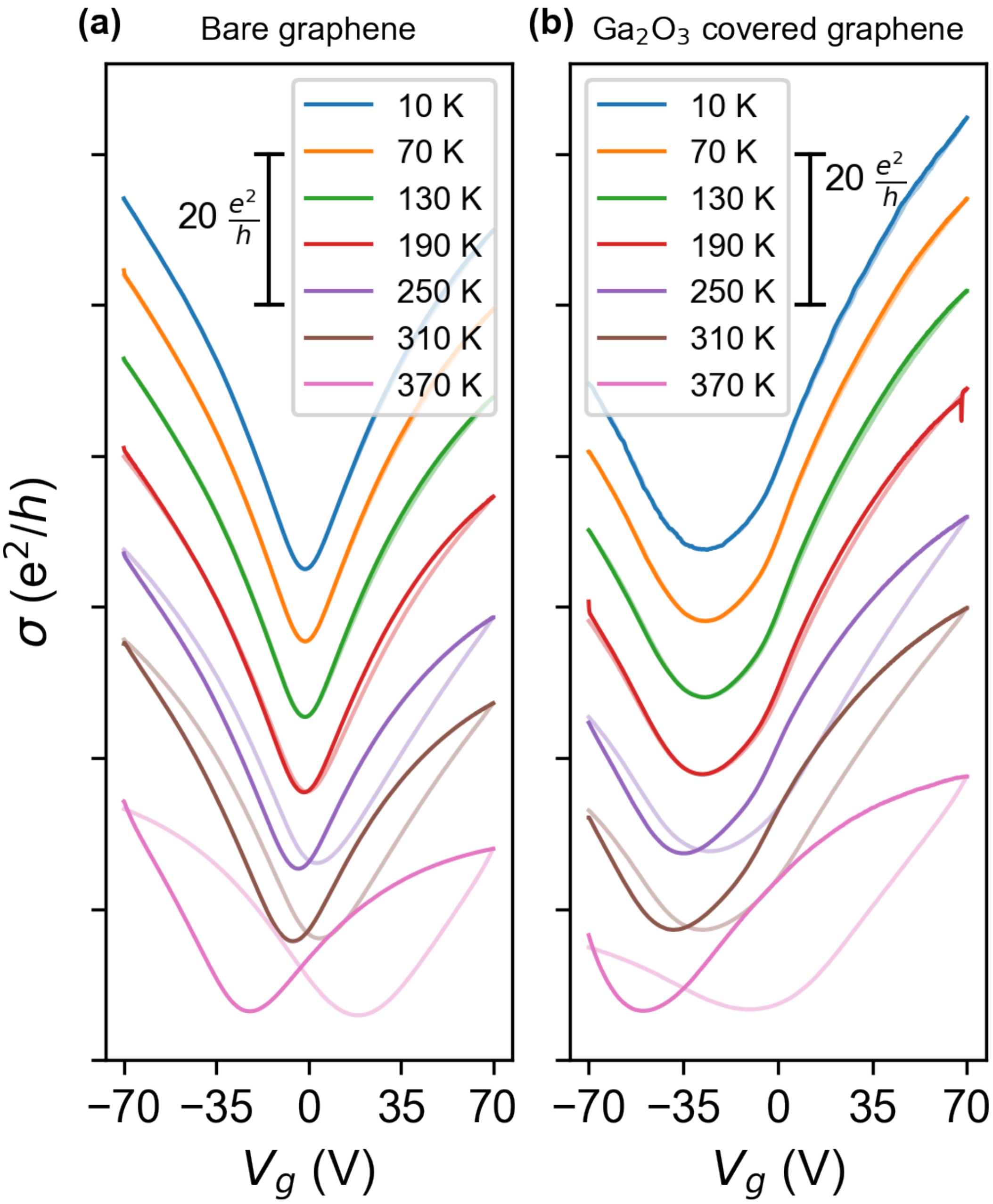

FIG. S2: Gate voltage-dependent conductivity $\sigma(V_g)$ at different temperatures for (a) bare graphene and (b) Ga_2O_3-covered graphene. The hysteresis becomes prevalent above 180 K. Dark colour indicates up-sweep, light indicates down-sweep.

TABLE S2: Intrinsic dielectric parameters used to compute ROP scattering.

Dielectric constants in units of ϵ_0 and frequencies in units of meV. For Ga_2O_3, ϵ_0 and is taken from Ref.[4] and ω_{TO1} is inferred from FTIR measurements in Ref.[5]

	SiO_2[6,7]	Ga_2O_3[4,5,8,9]	HfO_2[10]	Al_2O_3[6,7]	h-BN[7]
ϵ^0	3.9	10	17	12.53	5.09
ϵ^{int}	3.05	-	-	7.27	4.57
ϵ^{∞}	2.5	3.55	-	3.20	4.1
ω_{TO1}	55.6	67.7	40	48.18	97.4
ω_{TO2}	138.1	-	-	71.41	187.9

S8. Remote optical phonon (ROP) Scattering Model

According to the model described by the references,[6,10,11] when graphene is sandwiched between two different dielectrics, the optical phonon modes of the two dielectrics are modified to give rise to surface optical (SO) phonon modes. The electric field due to these modes may scatter carriers in an adjacent material, known as remote optical phonon scattering. The interface SO frequencies between two materials are calculated via a dielectric continuity equation

$$\sum \epsilon_{ox_i}(\omega) = 0 \tag{S21}$$

$$\epsilon_{ox_i}(\omega) = \epsilon_\infty + \sum_j^N f_j \frac{\omega_{TO}^{(j)\,2}}{\omega_{TO}^{(j)\,2} - \omega^2} \tag{S22}$$

where f_j is the strength of the dielectric contribution of the jth transverse optical (TO) mode for oxide i with N many TO modes. f_j corresponds to a difference between dielectric constants at different frequencies.

For example, for $N = 1$,

$$f_1 = \epsilon_{ox_i}^0 - \epsilon_{ox_i}^\infty \tag{S23}$$

Here $\epsilon_{ox_i}^0$ is the static dielectric constant and $\epsilon_{ox_i}^\infty$ is optical dielectric constant.

For $N = 2$,

$$f_j = \begin{cases} \epsilon_{ox_i}^{int} - \epsilon_{ox_i}^\infty & j = 2 \\ \epsilon_{ox_i}^0 - \epsilon_{ox_i}^{int} & j = 1 \end{cases} \tag{S24}$$

Here, $\epsilon_{ox_i}^{int}$ is the intermediate dielectric constant describing the dielectric response of the insulator at some intermediate frequency ω_{int} such that $\omega_{TO1} \leq \omega_{int} \leq \omega_{TO2}$.

For bare graphene on an oxide dielectric, eqn. S21 is modified to the following,

$$\epsilon_{ox_i}(\omega) + 1 = 0 \tag{S25}$$

The electron-ROP coupling strength for kth ROP mode resulting from the ith dielectric material is given by,

$$g_k = \hbar\omega_k \left(\frac{1}{\epsilon_{ox_i}^{(k+1)} + \epsilon_{ox_j}^{\infty}} - \frac{1}{\epsilon_{ox_i}^{(k)} + \epsilon_{ox_j}^{\infty}} \right) \tag{S26}$$

Here k corresponds to the index of the kth SO mode resulting from i^{th} material. k also relates the dielectric pairs $\{\epsilon_{ox_i}^{(k)}, \epsilon_{ox_i}^{(k+1)}\}$ that correspond to the TO mode pairs seen in Eq. (S24) ie. $\{\epsilon_{ox_i}^{0}, \epsilon_{ox_i}^{int}\}$ or $\{\epsilon_{ox_i}^{int}, \epsilon_{ox_i}^{\infty}\}$.

The various characteristic dielectric constants and relevant TO-mode frequencies for the various dielectric materials are mentioned in table S2. For Ga_2O_3, we assume $\epsilon_\infty = 3.55$, similar to measurements in non-amorphous Ga_2O_3 (α and β polymorphs[9])

Solving Eq. (S21), we find the resulting SO modes of various dielectric environments. Their corresponding electron-phonon coupling strengths are obtained from eqn. S26. The resultant ROP frequencies and the coupling strengths for various dielectric environment for graphene, e.g. bare graphene on SiO_2 Table S3, bare graphene on hBN Table S4, HfO_2-covered graphene on SiO_2 Table S5, and Ga_2O_3-covered graphene on SiO_2 Table S6.

TABLE S3: Calculated SO mode frequencies and coupling strengths for bare graphene on SiO_2

	$\hbar\omega_i$ (meV)	g_i (meV)
ω_1	61	2.6
ω_2	149	5.8

TABLE S4: Calculated SO mode frequencies and coupling strengths for bare graphene on hBN

	$\hbar\omega_i$ (meV)	g_i (meV)
ω_1	102	1.6
ω_2	197	3.3

TABLE S5: Calculated SO mode frequencies and coupling strengths for HfO_2-covered graphene on SiO_2

	$\hbar\omega_i'$ (meV)	g_i (meV)
ω_3	53	5.3
ω_1'	71	1.1
ω_2'	145	1.7

TABLE S6: Calculated SO mode frequencies and coupling strengths for Ga_2O_3-covered graphene SiO_2. Here ω_1', ω_2' are the perturbed SiO_2 modes, while ω_3 originates in Ga_2O_3.

	$\hbar\omega_i'$ (meV)	g_i (meV)
ω_1'	56	1.0
ω_3'	95	8.1
ω_2'	147	2.0

S9. Ga_2O_3 Atomic-force & Darkfield Microscopy

<table>
<tr><th>Sample</th><th>Microscopy</th><th>AFM</th></tr>
<tr><td>Sample 2 P01</td><td>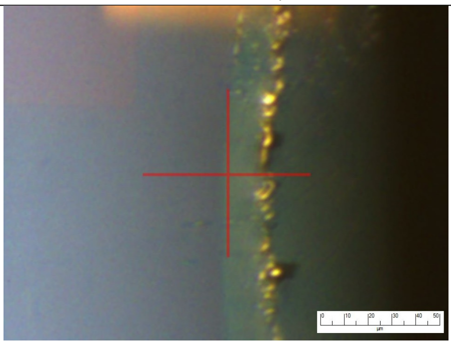</td><td>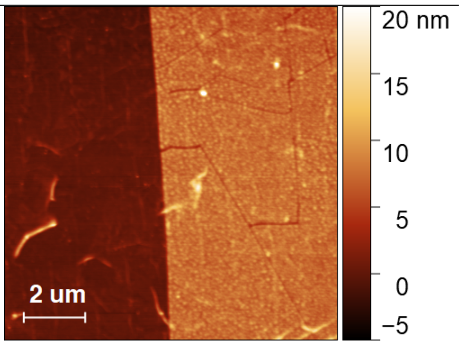
</td></tr>
<tr><td>Sample 2 P12</td><td>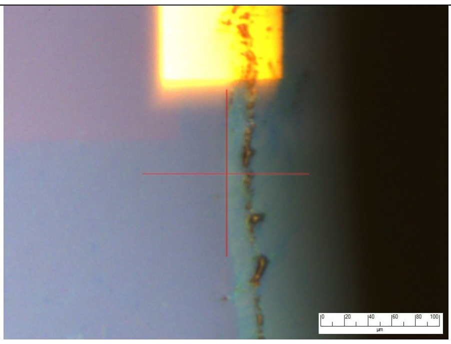</td><td>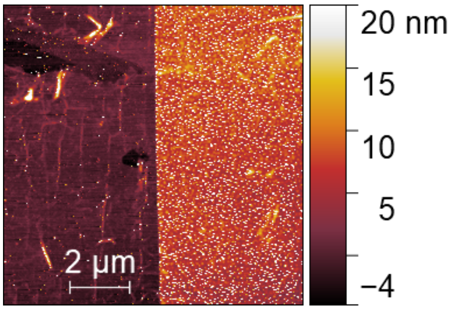
</td></tr>
<tr><td>Sample 3 P3</td><td>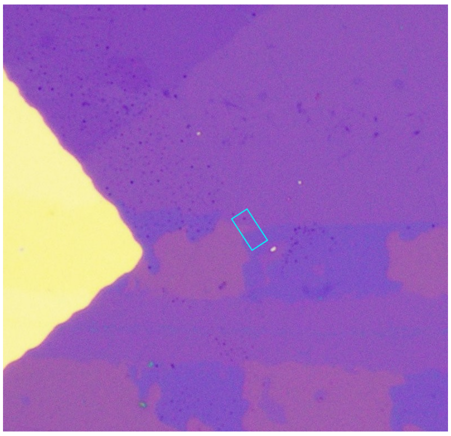</td><td>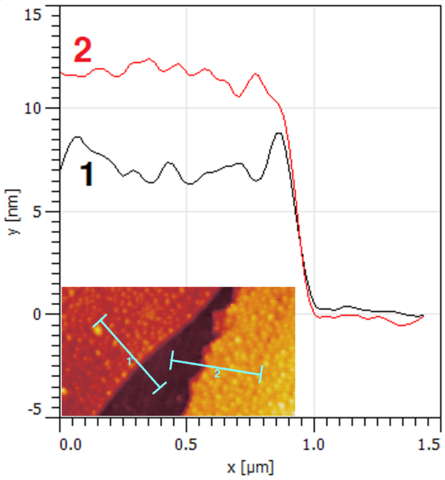

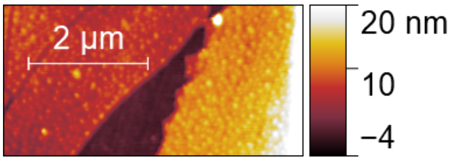
</td></tr>
</table>

Sample 3 P4	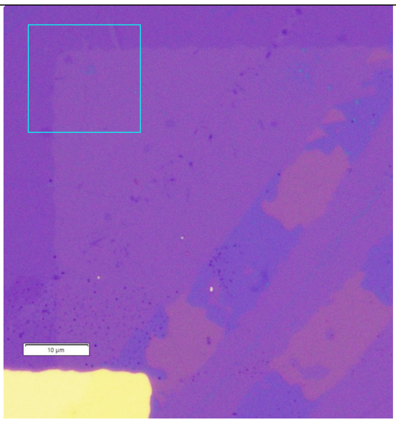	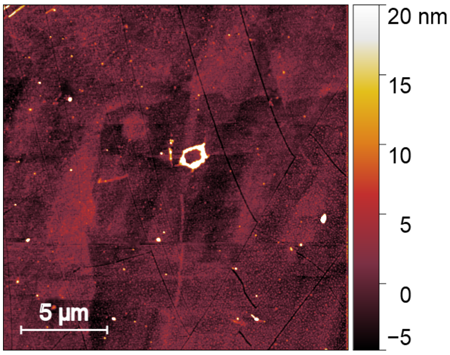
Sample 3 P5	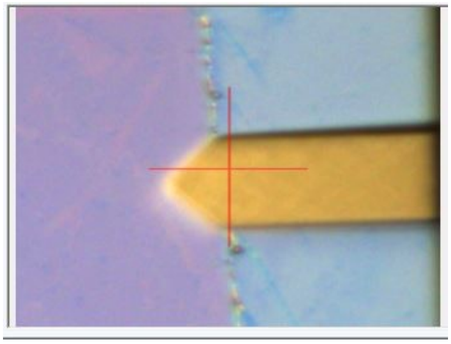	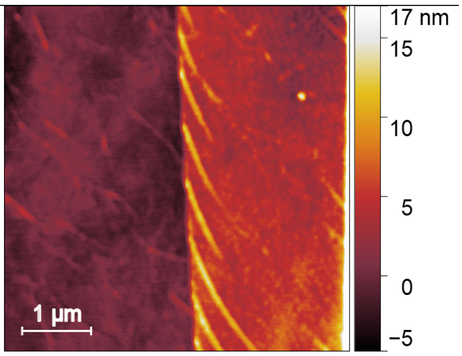

Sample	Microscopy	Darkfield
Dev4_03	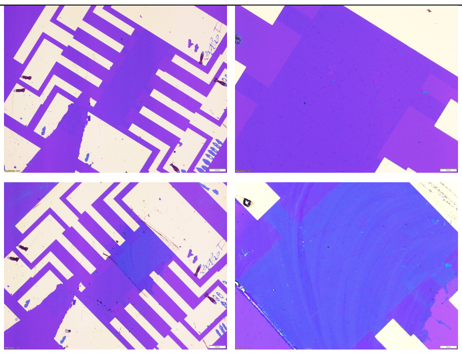	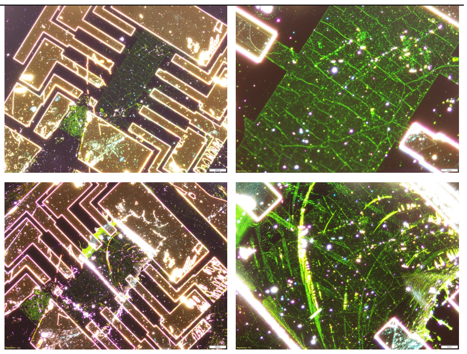
Dev4_04	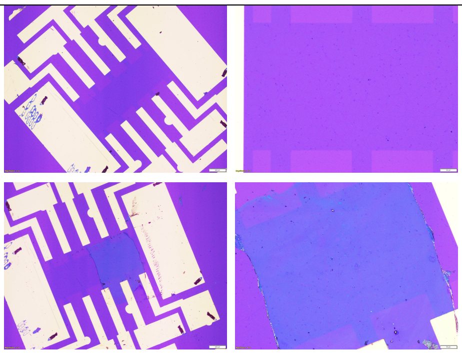	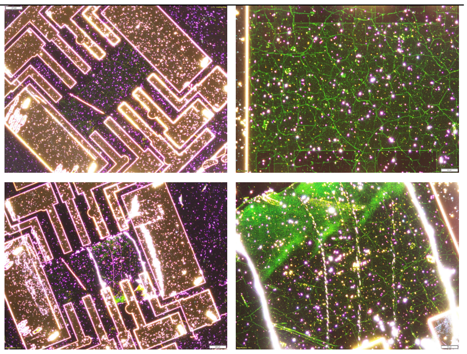

S10. Extracting ρ_{ROP} as a function of temperature

To analyse the phonon contributions to the resistivity of our devices, we wish to measure the temperature dependence of the resistivity at specific voltages away from the gate voltage of minimum conductivity $V_{g,min}$. However $V_{g,min}$ may drift with temperature giving rise to a spurious temperature dependence if it is not correctly determined. $V_{g,min}$ can be determined in a variety of ways, such as extrapolation of the linear slopes of $\sigma(V_g)$, or fitting the gate-voltage-dependent conductivity to a quadratic near the minimum. These may result in slightly different values which can change the result of the temperature dependent analysis.

We found the minimum conductivity σ_{min} using four different methods: (1) we extrapolated the linear behaviour of $\sigma(V_g)$ for $V_g < V_{g,min}$ and $V_g > V_{g,min}$ to their crossing point, and (2-4) fit $\sigma(V_g)$ to quadratics applied to data (2) $\sigma < 1.1\sigma_{min}$, (3) $\sigma < 1.5\sigma_{min}$, and (4) $\sigma < 2.0\sigma_{min}$.

We then compared the temperature dependent resistivities for positive and negative $V_g - V_{g,min}$ of the same magnitude. We found that method (4) gave the best agreement of the temperature-dependent resistivity for positive and negative $V_g - V_{g,min}$ which are expected to be similar.

Quad 1.1 fits very nicely to local minimum, however fails to capture the curvature between linear slopes like Quad 2.0. Lin gives a similar estimate for Bare, but can deviate from Quad fitting for Ga_2O_3.

To fit the intrinsic longitudinal acoustic phonon contributions to graphene's resistivity Eq. (S27) is used to apply a fit to both our Ga_2O_3-covered and bare samples. A subset of temperatures are used to fit the acoustic phonons, such that influences from localisation and ROP scattering (high temperature) and quantum corrections to the conductivity (low temperature) do not affect the fitting.

$$\rho_{LA} = \left(\frac{h}{e^2}\right)\frac{\pi^2 D_A^2 k_B^2}{2h^2\rho_s^2 v_s^2 v_F^2}T \tag{S27}$$

The fits are shown in Fig. S3.

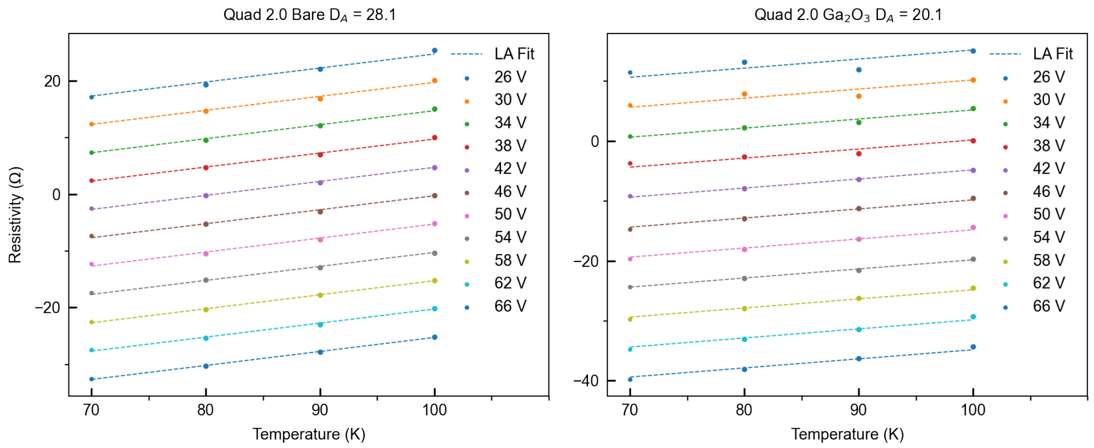

FIG. S3: Longitudinal acoustic (LA) fits for bare and Ga_2O_3

.

Here we used a temperature range of 70 K to 100 K to avoid the quantum corrections and ROP

contributions. Given that the remote phonon contributions to graphene's resistivity are expected to be smaller in the Ga_2O_3-covered graphene, we take the lower value of $D_A = 20.1 \pm 1.47$ eV found in the Ga_2O_3-covered graphene as the value for both systems when determining the ρ_{LA} to subtract from both bare and Ga_2O_3-covered Gr when analysing for ROP contributions. The value of $D_A = 20.1 \pm 1.47$ eV is consistent with other experiments in the literature.[10,12]

Lastly we propagate uncertainty in D_A through to the parameter obtained from the fitting to the ROP contribution to the resistivity. This is done by calculating the ρ_{ROP} contribution for each of the four methods to determine $V_{g,min}$, and for three values of D_A (18.6, 20.1, 21.6) eV. We use the standard deviation of these results to calculate the propogated uncertainty contributed from uncertainty in determining D_A and $V_{g,min}$, and combine it in quadrature with the fit uncertainty in ρ_{ROP} to provide the values listed. The propagated uncertainties are similar to in magnitude to the ρ_{ROP} fit uncertainties.

S11. Additional devices to assess reproduciblity of changes in transport properties after Ga_2O_3 passivation and ALD

A. Device fabrication and general properties

We have fabricated and measured an additional 60 devices (30 with Ga_2O_3 passivation, and 30 undergoing identical processing but without Ga_2O_3) to demonstrate the consistent effect of the addition of Ga_2O_3. These devices use the same fabrication processing as described in the main text, with the additional etching gap between device channels as described below, and larger sheets of Ga_2O_3. The devices are macroscopic (each channel is 100 μm x 120 μm and are passivated using Ga_2O_3 sheets 1 mm long. To compare devices undergoing passivation with bare graphene, we have designed the devices in matched pairs in close proximity (40 μm away) to each other.

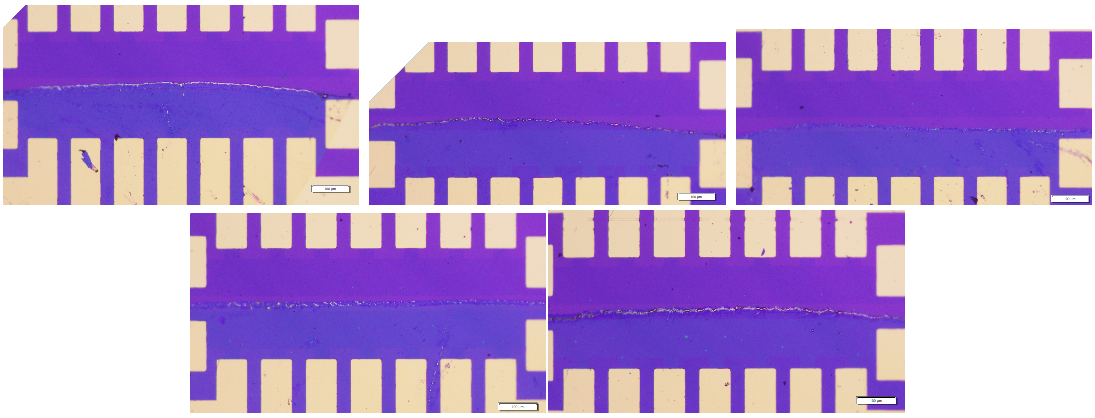

FIG. S4: Additional devices. Each group of devices consists of upper and lower graphene channels spaced 40 μm apart, with current electrodes at the ends and seven voltage proves allowing six channel sections ("devices" with dimensions 100 μm by 120 μm) to be probed independently on each channel. Ga_2O_3 was transferred on the lower channel of each device group, and the Al_2O_3 deposited by ALD over the entire structure. The micrographs above show the devices after Ga_2O_3 deposition but before Al_2O_3.

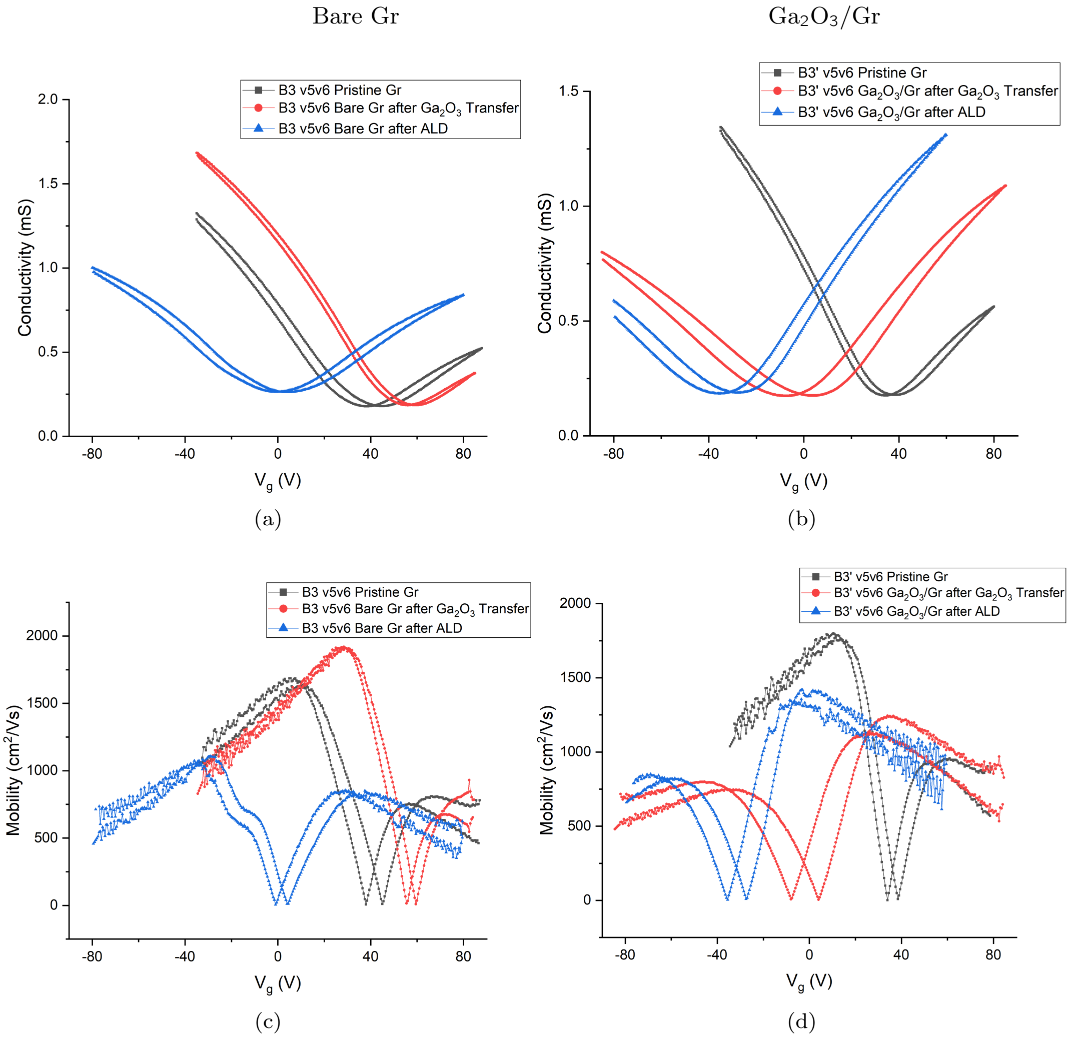

FIG. S5: Conductivity (a,b) and field-effect mobility (c,d) of a pair of representative devices, measured at room temperature in a vacuum probestation. (a,c) show the device which was not passivated by Ga_2O_3, while (c,d) show the Ga_2O_3 passivated device. The legend indicates the measurements performed before Ga_2O_3 passivation, after passivation and after ALD.

B. Statistical analysis

1. Ga_2O_3 transfer

	Bare Gr	Ga_2O_3/Gr Side
$\mu_e{}^{\text{After}}$ / $\mu_e{}^{\text{Before}}$	0.96±0.12	1.45±0.15
$\mu_h{}^{\text{After}}$ / $\mu_h{}^{\text{Before}}$	1.18±0.08	0.48±0.04

TABLE S7: Ratio of mobilities (after:before) Ga_2O_3 deposition. Electron (μ_e) and hole (μ_h) mobilities are shown for bare (control) and Ga_2O_3 passivated sides of device.

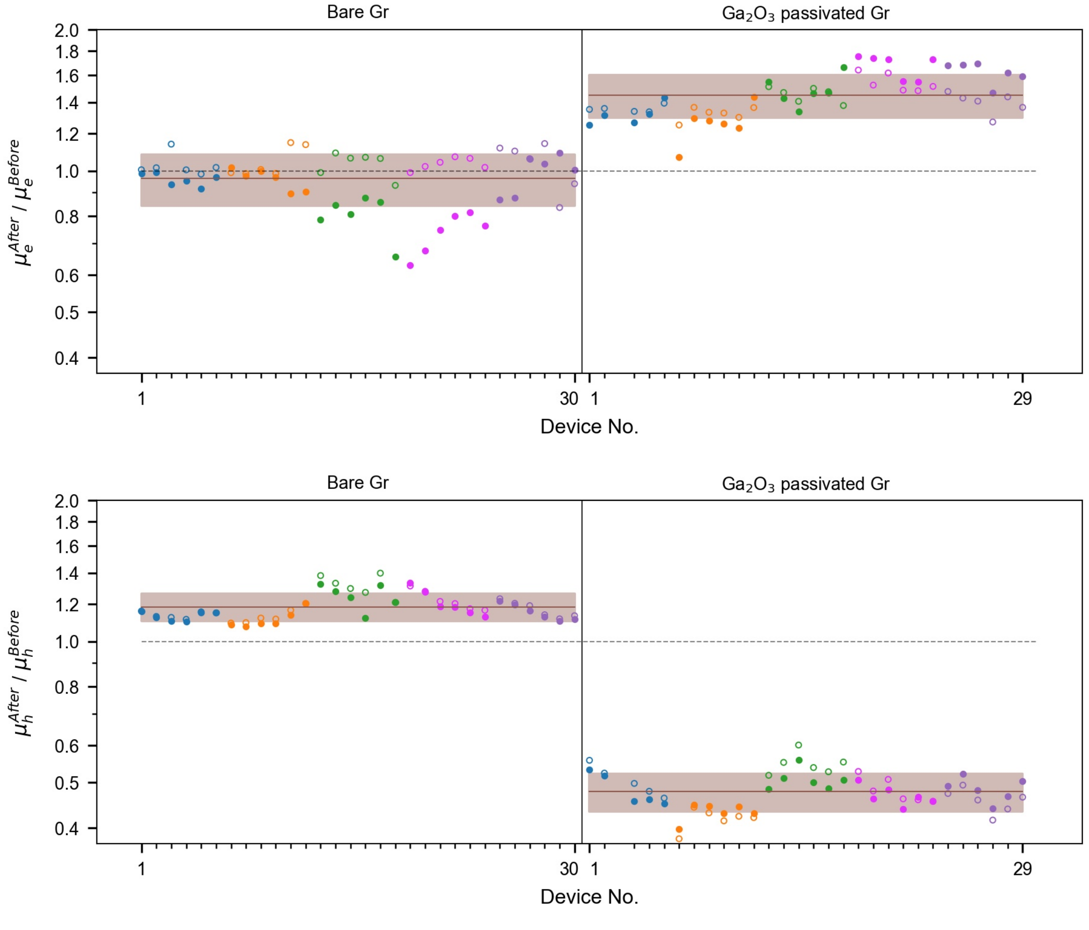

FIG. S6: Changes to (a) μ_e and (b) μ_h mobility measured at room temperature after Ga_2O_3 transfer step on bare and Ga_2O_3 passivated areas. Filled (open) circles correspond to voltage sweep direction in the -ve to +ve (+ve to -ve) direction. Colour indicates devices that are (i) physically connected to each other or (ii) in local proximity as a pair of bare-Ga_2O_3 devices as also indicated by device number.

	Bare Gr	Ga_2O_3 Gr
$\mu_e{}^{\text{After}}$ / $\mu_e{}^{\text{Before}}$	1.17±0.28	1.12±0.11
$\mu_h{}^{\text{After}}$ / $\mu_h{}^{\text{Before}}$	0.57±0.08	1.06±0.14

TABLE S8: Ratio of mobilities (after:before) ALD Al_2O_3 deposition. Electron (μ_e) and hole (μ_h) mobilities are shown for bare (control) and Ga_2O_3 passivated sides of device.

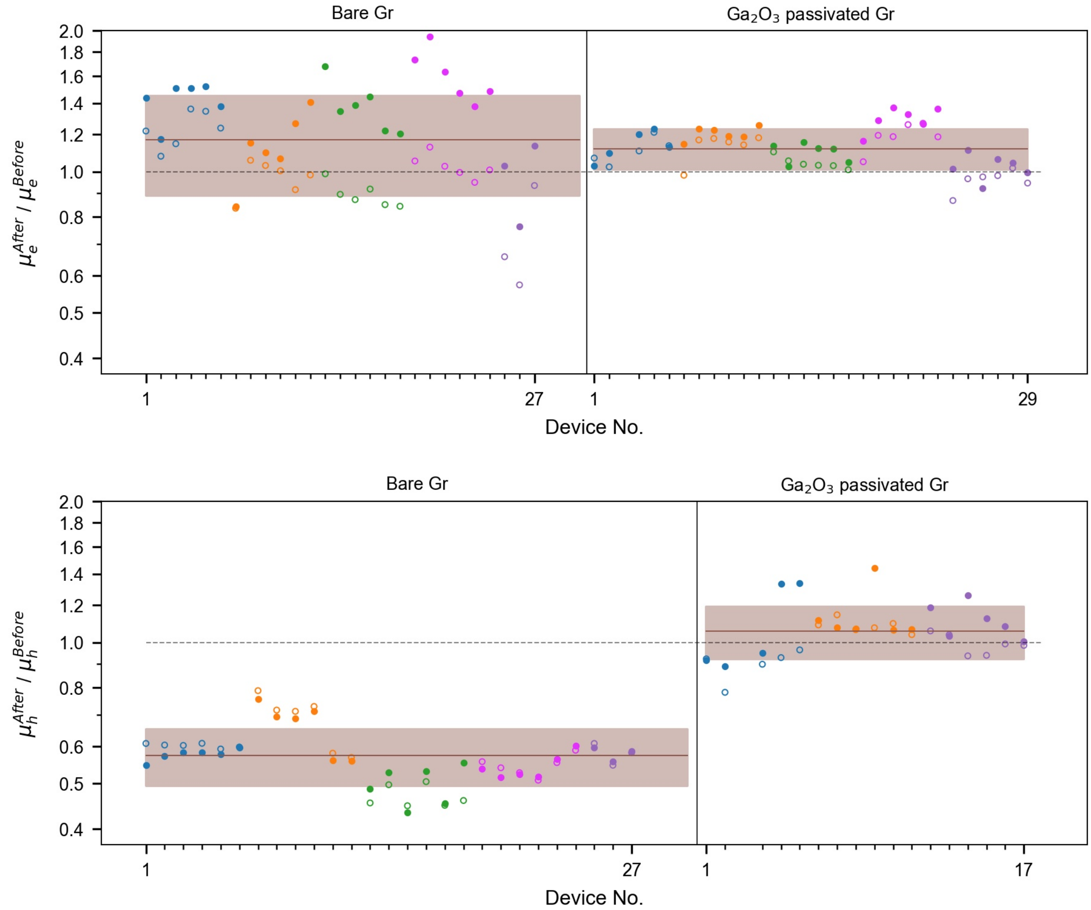

FIG. S7: Changes to (a) μ_e and (b) μ_h mobility measured at room temperature after the step of ALD deposition of Al_2O_3 on bare and Ga_2O_3 passivated areas. Filled (open) circles correspond to voltage sweep direction in the -ve to +ve (+ve to -ve) direction. Colour indicates devices that are (i) physically connected to each other or (ii) in local proximity as a pair of bare-Ga_2O_3 devices. (ii) is also indicated by device number in all cases with the exception of μ_h where two devices groups were too doped to properly measure hole mobility.

S12. References

[*] Corresponding authors: semonti.bhattacharyya@monash.edu and michael.fuhrer@monash.edu
[†] Corresponding author: semonti.bhattacharyya@monash.edu

[1] Xiaohui Tang, Nicolas Reckinger, Olivier Poncelet, Pierre Louette, Ferran Ureña, Hosni Idrissi, Stuart Turner, Damien Cabosart, Jean-François Colomer, Jean-Pierre Raskin, Benoit Hackens, and Laurent A. Francis, "Damage evaluation in graphene underlying atomic layer deposition dielectrics," Sci. Rep. **5**, 13523 (2015).

[2] Matthias Wurdack, Tinghe Yun, Eliezer Estrecho, Nitu Syed, Semonti Bhattacharyya, Maciej Pieczarka, Ali Zavabeti, Shao-Yu Chen, Benedikt Haas, Johannes Müller, Mark N. Lockrey, Qiaoliang Bao, Christian Schneider, Yuerui Lu, Michael S. Fuhrer, Andrew G. Truscott, Torben Daeneke, and Elena A. Ostrovskaya, "Ultrathin Ga_2O_3 Glass: A Large-Scale Passivation and Protection Material for Monolayer WS2," Adv. Mater. **33**, 2005732 (2021).

[3] Shaffique Adam, E. H. Hwang, V. M. Galitski, and S. Das Sarma, en"A self-consistent theory for graphene transport," Proc. Natl. Acad. Sci. U. S. A. **104**, 18392–18397 (2007).

[4] M. Passlack, N. E. J. Hunt, E. F. Schubert, G. J. Zydzik, M. Hong, J. P. Mannaerts, R. L. Opila, and R. J. Fischer, "Dielectric properties of electron-beam deposited Ga_2O_3 films," Appl. Phys. Lett. **64**, 2715–2717 (1994).

[5] A. Ortiz, J. C. Alonso, E. Andrade, and C. Urbiola, "Structural and Optical Characteristics of Gallium Oxide Thin Films Deposited by Ultrasonic Spray Pyrolysis," Journal of The Electrochemical Society **148**, F26 (2001).

[6] Massimo V. Fischetti, Deborah A. Neumayer, and Eduard A. Cartier, "Effective electron mobility in Si inversion layers in metal–oxide–semiconductor systems with a high- insulator: The role of remote phonon scattering," J. Appl. Phys. **90**, 4587–4608 (2001).

[7] Zhun-Yong Ong and Massimo V. Fischetti, "Theory of interfacial plasmon-phonon scattering in supported graphene," Phys. Rev. B **86**, 165422 (2012).

[8] M. Passlack, E. F. Schubert, W. S. Hobson, M. Hong, N. Moriya, S. N. G. Chu, K. Konstadinidis, J. P. Mannaerts, M. L. Schnoes, and G. J. Zydzik, "Ga_2O_3 films for electronic and optoelectronic applications," J. Appl. Phys. **77**, 686–693 (1995).

[9] S. J. Pearton, Jiancheng Yang, Patrick H. Cary, F. Ren, Jihyun Kim, Marko J. Tadjer, and Michael A. Mastro, "A review of Ga_2O_3 materials, processing, and devices," Applied Physics Reviews **5**, 011301 (2018).

[10] K. Zou, X. Hong, D. Keefer, and J. Zhu, "Deposition of High-Quality HfO_2 on Graphene and the Effect of Remote Oxide Phonon Scattering," Phys. Rev. Lett. **105**, 126601 (2010).

[11] S. Fratini and F. Guinea, "Substrate-limited electron dynamics in graphene," Phys. Rev. B **77**, 195415 (2008).

[12] J.-H. Chen, C. Jang, S. Adam, M. S. Fuhrer, E. D. Williams, and M. Ishigami, "Charged-impurity scattering in graphene," Nat. Phys. **4**, 377–381 (2008).
